# In Vitro Assessment of Poly-N-Vinylpyrrolidone/Acrylic Acid Nanoparticles Biocompatibility in a Microvascular Endothelium Model

**DOI:** 10.3390/ijms232012446

**Published:** 2022-10-18

**Authors:** Aikaterini Berdiaki, Andrey N. Kuskov, Pavel P. Kulikov, Lydia-Nefeli Thrapsanioti, Eirini-Maria Giatagana, Polychronis Stivaktakis, Mikhail I. Shtilman, Aristidis Tsatsakis, Dragana Nikitovic

**Affiliations:** 1 Laboratory of Histology-Embryology, School of Medicine, University of Crete, 71003 Heraklion, Greece; 2 Department of Technology of Chemical Pharmaceutical and Cosmetic Substances, D. Mendeleev University of Chemical Technology of Russia, 125047 Moscow, Russia; 3 Centre for Strategic Planning of FMBA of Russia, 119121 Moscow, Russia; 4 Laboratory of Toxicology, School of Medicine, University of Crete, 71003 Heraklion, Greece; 5 Department of Biomaterials, Mendeleev University of Chemical Technology of Russia, Miusskaya sqr. 9, 125047 Moscow, Russia

**Keywords:** poly-N-vinylpyrrolidone, acrylic acid, amphiphilic polymer, nanoparticle, endothelial cells, assessment, viability, immunological activation, fluorescent probes

## Abstract

An amphiphilic copolymer of N-vinyl-2-pyrrolidone and acrylic acid—namely, p(VP-AA)-OD6000 (p(VP-AA))—was synthesized to prepare p(VP-AA) nanoparticles (NPs). Furthermore, the copolymer was linked with CFSE, and the so-prepared nanoparticles were loaded with the DiI dye to form D nanoparticles (DNPs). In this study, as demonstrated by immunofluorescence microscopy, immunofluorescence, and confocal microscopy, DNPs were readily taken up by human microvascular endothelial cells (HMEC-1) cells in a concentration-dependent manner. Upon uptake, both the CFSE dye (green stain) and the DiI dye (red stain) were localized to the cytoplasm of treated cells. Treatment with p(VP-AA) did not affect the viability of normal and challenged with LPS, HMEC-1 cells at 0.010 mg/mL and induced a dose-dependent decrease of these cells’ viability at the higher concentrations of 0.033 and 0.066 mg/mL (*p* ≤ 0.01; *p* ≤ 0.001, respectively). Furthermore, we focused on the potential immunological activation of HMEC-1 endothelial cells upon p(VP-AA) NPs treatment by assessing the expression of adhesion molecules (E-Selectin, ICAM-1, and V-CAM). NPs treatments at concentrations utilized (*p* = NS) did not affect individual adhesion molecules’ expression. p(VP-AA) NPs do not activate the endothelium and do not affect its viability at pharmacologically relevant concentrations.

## 1. Introduction

Nanoparticles (NPs) are materials exhibiting specific physicochemical properties which bestow unique abilities to interact with biological systems. Characteristics in morphology, shapes, or sizes are used to classify NPs. Modifications in NP structure can bestow specific affinities when interacting with cells and tissues. Indeed, their application in biomedicine is rapidly increasing due to their ability to deliver in a targeted manner a multitude of drugs giving solutions to unmet medical needs [1,2,3,4].

For several years, we have focused on synthesizing amphiphilic derivatives of poly-N-vinylpyrrolidone (Amph-PVP) composed of various molecular weight polymeric hydrophilic fragments linked into hydrophobic n-alkyl chains of varying lengths [5,6,7]. Notably, Amph-PVPs can be used to modify liposome membranes to increase their stability and efficiency [8] or can self-assemble in aqueous media, achieving an efficient entrapment of many hydrophobic drugs [9,10,11]. Moreover, the initial evaluation of Amph-PVP NPs, in vitro and in vivo, demonstrated satisfactory stability in biological media and biocompatibility [12,13,14], verifying their utilization directly. Recently, in vivo, Amph-PVP NPs loaded with an anti-inflammatory drug, indomethacin, exhibited superior anti-inflammatory activity and a safe gastrointestinal profile. Indeed, these results suggest that Amph-PVPs present a therapeutic alternative for the currently available non-steroidal anti-inflammatory drug (NSAID) administration [15]. Notably, the utilization of some poly-N-PVP-based carriers has been approved for clinical studies [16].

Derivatives of copolymers of N-vinyl-2-pyrrolidone with acrylic acid p(VP-AA)at were previously utilized as components of hydrogels crosslinked using ethylene glycol dimethacrylate (EGDMA) [17], alumina nanoparticles (Al₂O₃ NPs) as the inorganic cross-linking agents [18], or alpha-tricalcium phosphate (α-TCP) cement [19]. Likewise, a hybrid “protein-copolymer” microbubble shell with a complex of bovine serum albumin and an amphiphilic copolymer of p(VP-AA) demonstrated potential as advanced ultrasound contrast agents with satisfactory in vitro biocompatibility [20]. Moreover, in hepatoma and normal keratinocyte, HaCaT cells, the extracts of EGDMA-linked p(VP-AA) hydrogels were shown to be non-toxic at high concentrations [17]. Furthermore, in a mouse melanoma model, p(VP-AA) copolymers linked to peptide-based vaccine prototypes exhibited a potent immune-stimulating effect indicating that they can be utilized as an adjuvant for the immunological system [21]. Indeed, they may improve the vaccine potential of antigens by facilitating their delivery and reducing the booster doses of vaccines [21]. We recently synthesized and modified with acrylic acid PVP copolymers to prepare nanoparticles (NPs) [22].

The delivery of nanocarrier-drug complexes is perpetrated via different routes of administration, such as injection into blood vessels, inhalation, and oral and transdermal application [23]. p(VP-AA) NPs were shown to be taken up in vivo in a retina model and in vitro by blood cells [22]. Independently of the application route, the NPs will enter the circulatory system after being taken up by blood and/or lymphatic vessels. In all cases, the endothelial cells (EC), which coat blood/lymphatic vessels, will be exposed to NPs. Exposure can disrupt these cells’ homeostasis and immunological response [24,25].

Notably, the ECs are a vital component of the semi-selective barrier, which separates the parenchyma of peripheral organs and tissues from the blood. Indeed, the ECs are a crucial effector in maintaining the body’s vascular homeostasis as solutes and immune cells have to traverse the endothelium to leave or enter the bloodstream to preserve homeostasis [26]. Furthermore, Ecs are heterogeneous and exhibit, albeit slight, discrete morphological, physiological, and phenotypic differences in the various compartments of the vascular system and between arteries and veins, facilitating their respective functions in specific vascular areas [27]. Notably, microvascular Ecs uniquely support communication through the vascular barrier, modulating immune reactions, secretion, and metabolism of bioactive molecules [28]. Therefore, the microvascular blood-vessel interface is distinctive in enabling homeostasis and regulating essential functions of tissues/organs [29].

Ecs participate in the inflammatory response by changing function and morphology, denominated as endothelial cell activation [30]. These changes lead to a reduction in vascular integrity, an increase in the expression of adhesion molecules and cytokine production propagating inflammation, and an enhancement of human leukocyte antigen (HLA) molecules [31]. The increase of adhesion molecules, including ICAM-1 (intercellular adhesion molecule-1), VCAM-1 (vascular cell adhesion molecule-1), and E-Selectin, facilitates the adherence of leukocytes on the endothelium and their subsequent transmigration to the sub-endothelium [32,33]. In addition, the production of cytokines, including interleukins and tumor necrosis factors, regulates the immune response [31,33]. The above enhances the risk of thrombocytic events [33]. Therefore, a thorough investigation of nanoparticles with the endothelium is an obligatory step in NPs’ biocompatibility evaluation.

In the present study, we synthesized fluorescently labeled with CFSE and DiI, p(VP-AA) NPs, namely DNPs. The labeled p(VP-AA) NPs were utilized to study these NPs’ uptake, intracellular localization, and effects on endothelial cell viability. Since p(VP-AA) polymers were shown to affect immune system activation, we investigated their effects on endothelial cell activation by assessing the expression of specific adhesion molecules.

## 2. Results and Discussion

Due to their capability to increase drug bioavailability, targeting, and pharmaceutical efficiency while simultaneously limiting side toxicity, NPs are widely studied and increasingly applied in the biomedical sector [34]. Despite the global use of different nano-scaled polymeric drug carriers for research, more detailed information concerning their biological effects, toxicity, and safety is still required [35,36]. In our previous study, we determined that the NPs composed of Amph-PVP NPs (2000, 4000, 6000, 8000, and 12,000 Da) did not affect the growth of HMEC-1 endothelial cells up to the high concentration of 0.066 mg/mL exhibiting outstanding blood biocompatibility [14]. In this study, and based on our previous studies, we chose the 6000 Da polymer molecular weight as the most suitable for producing a copolymer with acrylic acid (p(VP-AA)).

### 2.1. Preparation and Characterization of p(VP-AA) NPs Carrying Fluorophores

**Figure 1:**
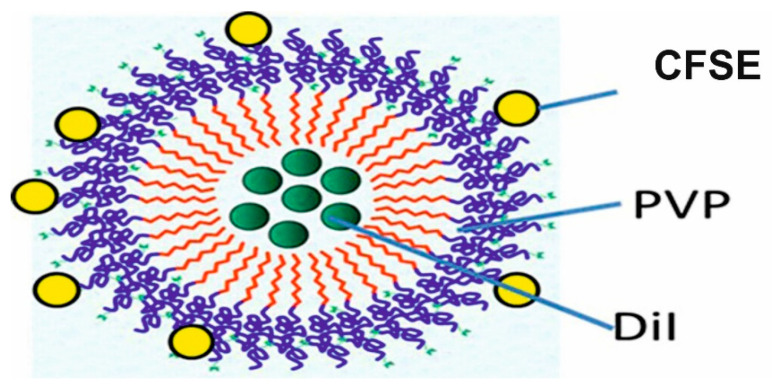
Structure of DNPs. DiI pigment molecules are incorporated into p(VP-AA) NPs, and CFSE (linked CFSE) molecules are attached to p(VP-AA) NPs.

**Figure 2:**
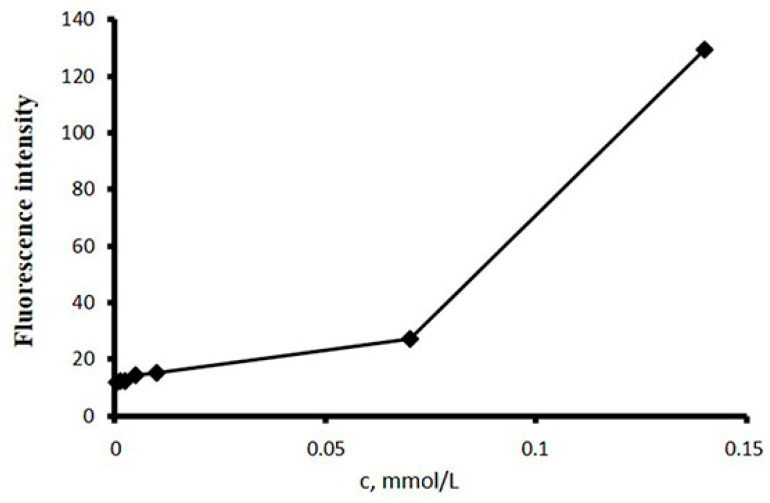
Fluorescence intensity of the p(VP-AA)-ODM solution with DPHT at 25 °C.

The synthesis and characterization of these NPs were performed as previously shown [22]. Furthermore, the fluorescent labeling of p(VP-AA) NPs was conducted to enhance the evaluation of their interaction with endothelial cells in vitro, such as precisely determining their location during cell uptake and quantifying their entry into the cytoplasm. Thus, the p(VP-AA) were coupled and labeled with the specific fluorescent CFSE and DiI molecules to create DNPs. Specifically, CFSE was covalently linked to p(VP-AA), whereas DiI was loaded to the hydrophobic NPs’ cavity (Figure 1). The concentration of CFSE was 3 mg/g, while the concentration of DiI was 11 mg/g. The CAC of the synthesized p(VP-AA) copolymer was determined in the presence of DPHT. This approach demonstrated that the CAC is 0.073 mmol/L (Figure 2). The mean particle size of DNPs was 172 nm (Table 1).

Notably, the zeta potential of NPs designated for drug delivery applications is a critical parameter that affects both their physical stability and the in vivo effects when administered [37]. Indeed, previously, it was demonstrated that a suitable surface charge could enhance the carrier release profile at specific sites as well as improve dosage efficiency. For low molecular-weight surfactants, pure electric stabilization is reflected by high absolute ZP values [38]. In contrast, for high molecular-weight surfactants (e.g., amphiphilic polymers), stabilization is provided through a multitude of mechanisms (e.g., steric stabilization) and not uniquely reflected by the absolute value of ZP [37,39,40,41,42,43] The ZP measurements for the p(VP-AA) nanoparticles are presented in Table 1. All nanoparticles were found to have slightly negative surface charges ranging from −15 to −18 mV.

In our previous study [44], the outcomes of several independent experiments demonstrate good thermodynamic and kinetic stability, i.e., low CMCs and NP size stability against various destabilizing factors (vide infra), and allows us to conclude that the low ZP values measured for the NPs cannot be correlated to the instability of the colloidal dispersions or aggregation.

**Figure 3:**
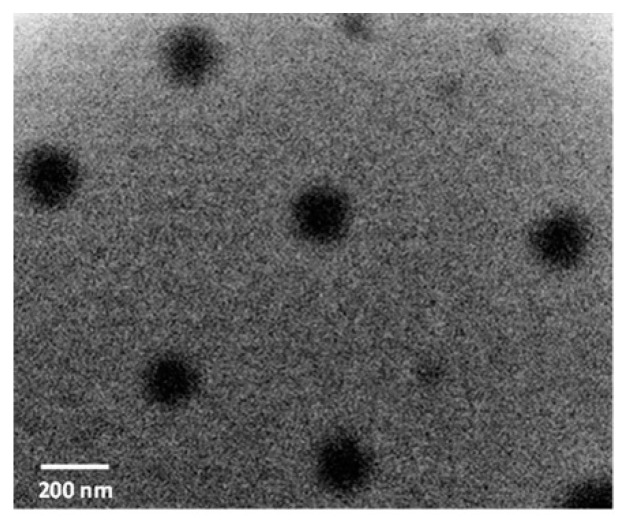
Hollow p(VP-AA) TEM micrograph showing their size and morphology. The images confirm the spherical shape of the NPs. Scale bar: 200 nm.

As was previously shown [44], the amphiphilic polymers can spontaneously self-assemble into nano-scaled, core-shell, spherically shaped particles. In this study, the self-assembled amphiphilic p(VP-AA) micellar NPs were successfully prepared using the emulsification method followed by solvent evaporation. This fabrication approach was previously found to be the best for the simple preparation of homogenously sized nanoparticles of amphiphilic polymers [44]. The average sizes of the resulting NPs, size distribution, and surface charge were determined by dynamic light scattering and are presented in Table 1. In the current study, though, it was determined by DLS that amphiphilic p(VP-AA) polymers self-assemble into NPs with narrow (unimodal) size distribution. The mean hydrodynamic diameter of the nanoparticles formed was less than 200 nm, which is suitable for creating nano-scaled drug delivery systems. The spherical morphology and the nano-scale size of the prepared p(VP-AA) polymeric particles were also visualized and confirmed by transmission electron microscopy (Figure 3).

### 2.2. Effect of p(VP-AA) NPs and DNPs on Healthy and Challenged with LPS HMEC-1 Cell Viability

**Figure 4:**
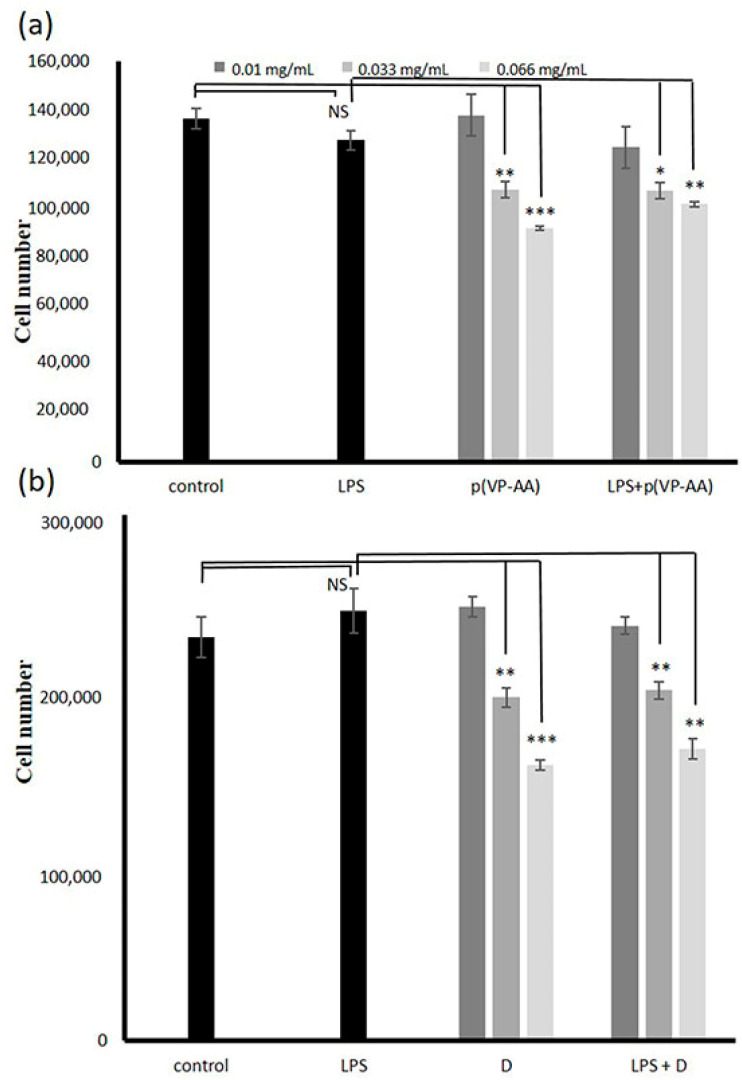
Effect of p(VP-AA) and DNPs on the viability of basal and activated HMEC-1 cells. (**a**) Concentration-dependent effect of p(VP-AA) NPs on HMEC-1 cells’ viability. Basal state and exposed to LPS cells were cultured in 96-well plates and treated with p(VP-AA) NPs for 48 h; (**b**) Concentration-dependent effect of DNPs (D) on HMEC-1 cells’ viability. Basal state and exposed to LPS cells were cultured in 96-well plates and treated with DNPs for 48 h. The results represent the average of three separate experiments. Statistical significance: * *p* ≤ 0.05, ** *p* ≤ 0.01, *** *p* ≤ 0.001, NS = not significant compared to respective controls.

In this study, we evaluated the effect of p(VP-AA) NPs on endothelial cells’ viability. Previously, extracts of p(VP-AA) hydrogels were shown to be non-toxic in human keratinocytes (HaCaT) and human hepatoma (HepG2) cell models [17]. Moreover, we determined that p(VP-AA) NPs labeled with CFSE are readily taken up by blood cells and did not induce hemolysis [22]. The effect of p(VP-AA) NPs and DNPs on HMEC-1 EC viability was assessed using the MTT assay as suggested by the manufacturer. Seeding cells at 8000 cells/well was suitable for cell growth experiments [11]. HMEC-1 cells were treated with NPs at the medium 0.010, high 0.033, and very high 0.066 mg/mL concentrations. No effect on ECs’ viability was determined at low concentrations below 0.010 mg/mL (Figure S1). Treatment with p(VP-AA) induced a decrease in cell viability at the higher concentrations of 0.033 and 0.066 mg/mL (*p* ≤ 0.01; *p* ≤ 0.001, respectively), as shown in Figure 4a. The therapeutic range of a vast majority of drugs is well below 0.001 mg/mL in blood plasma, as determined when evaluating approximately 1000 drugs and other xenobiotics [45]. Indeed, drug plasma concentration in the micromolar/L range is an exception [45]. Therefore, our results suggest that the safe concentrations of p(VP-AA) NPs regarding interaction with ECs fall well within the therapeutic range of most drugs [45].

Under pathological conditions, the ECs acquire the activated phenotype. It is widely acknowledged that an immune response of EC cells is induced upon exposure of endothelial cells to LPS and subsequent binding of LPS to the TLR4 receptor [46]. Furthermore, the binding of LPS to TLR4 initiates downstream phosphokinase activation and the phosphorylation of the cytoplasmic NF-κβ. The phosphorylated NF-κβ translocates to the nucleus, where it engages in the transcriptional regulation of pro-inflammatory molecules, which are, among others, correlated to the coagulation process [47,48]. As previously described, a model of activated endothelium was generated by challenging HMEC-1 cells with LPS for 2 h [11,14]. Exposing immunologically activated HMEC-1 cells to p(VP-AA) resulted in decreased cell viability at the concentrations of 0.033 and 0.066 mg/mL (*p* ≤ 0.05; *p* ≤ 0.01, respectively), similar to the exposure of basal state cells. These data suggest that p(VP-AA) NPs can be utilized in the normal therapeutic range [45] under pathological conditions. In control experiments, p(VP-AA) NPs did not affect Nf-κβ activation.

Furthermore, we evaluated the effects of labeled p(VP-AA) NPs, on HMEC-1 cells’ viability. Likewise, DNPs induced a dose-dependent decrease in these cells’ viability at the higher concentrations of 0.033 and 0.66 mg/mL (*p* ≤ 0.01; *p* ≤ 0.001, respectively) (Figure 4b), whereas at low and medium concentrations, these cells’ growth was not affected (*p* = NS). Suppression of activated HMEC-1 cells’ viability was also identified after treating cells with DNPs at the higher concentrations of 0.033 and 0.066 mg/mL (*p* ≤ 0.05; *p* ≤ 0.01, respectively), as shown in Figure 4b These results indicate that p(VP-AA) NPs inhibit the viability of HMEC-1 cells at higher concentrations. The effects of DNPs did not differ compared to p(VP-AA) NPs. Notably, the inhibitory effects were similar under basal and activated epithelium conditions. These results demonstrate that endothelium activation does not enhance the viability-suppressing effect of p(VP-AA) NPs.

### 2.3. Effect of p(VP-AA) NPs on Normal and Activated with LPS HMEC-1 Cell Growth

**Figure 5:**
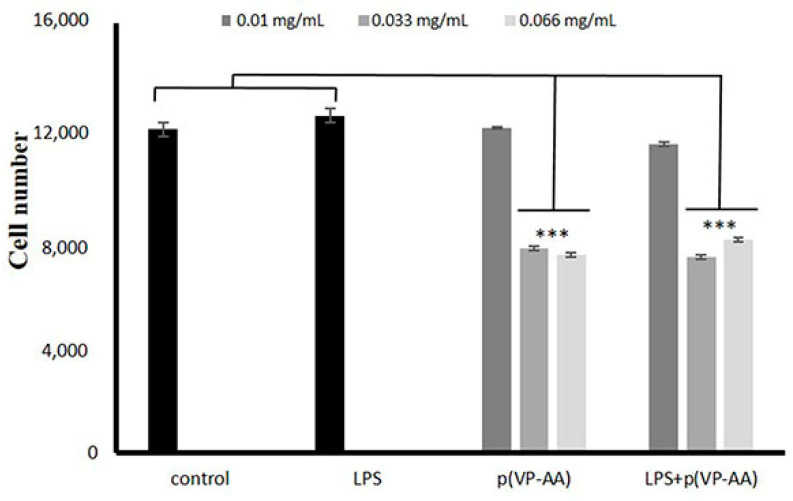
Effect of p(PV-AA) NPs of HMEC-1 cells’ growth. Basal state and activated with LPS HMEC-1 cells were cultured in 96-well plates and treated with p(PV-AA) NPs for 48 h. Cell number was determined utilizing the CyQuant Proliferation assay. The results represent the average of three separate experiments. Statistical significance: *** *p* ≤ 0.001 compared to controls.

Notably, ECs control thrombosis and thrombolysis, participate in interactions of leukocytes and platelets with the vessel wall, determine the contraction and dilation of blood vessels, and exhibit a role in organogenesis and regeneration [27,49,50,51]. Thus, their ability to proliferate vigorously correlates to maintaining homeostasis and forming new blood vessels [52]. Therefore, we evaluated the effect of p(VP-AA) NPs on EC growth. For this purpose, we utilized the CyQuant cell proliferation assay as instructed by the manufacturer. Specifically, under basal conditions, p(VP-AA) NPs strongly inhibited HMEC-1 cells’ growth ability at the higher concentrations of 0.033 and 0.066 mg/mL (*** *p* ≤ 0.001), as shown in Figure 5. This effect was duplicated when challenged with LPS; HMEC-1 cells were exposed to p(VP-AA) NPs. Thus, substantial inhibition of growth was likewise evident at the higher concentrations of 0.033 and 0.066 mg/mL (*p* ≤ 0.001) (Figure 5). These data demonstrate that in the normal therapeutic range, NPs do not exert adverse effects on EC growth [45,53].

In separate experiments utilizing CyQuant assay, we determined the half maximal inhibitory concentration (IC50) values when treating normal (basal levels) or LPS-activated endothelial cells with 0, 1, 10, 25, 50, 100, and 500 mg/mL p(PV-AA) NPs. Specifically, the x-y axis was plotted, and the data were fitted with a straight line (linear regression). IC50 value was then estimated using the fitted line, i.e., Y = a * X + b, IC50 = (50 − b)/a (Figure S2). The IC50 value for basal levels was 468.14 mg/mL and for LPS-activated, 465.52 mg/mL (Figure S2).

### 2.4. Uptake of DNPs by Microvascular Endothelial Cells

**Figure 6:**
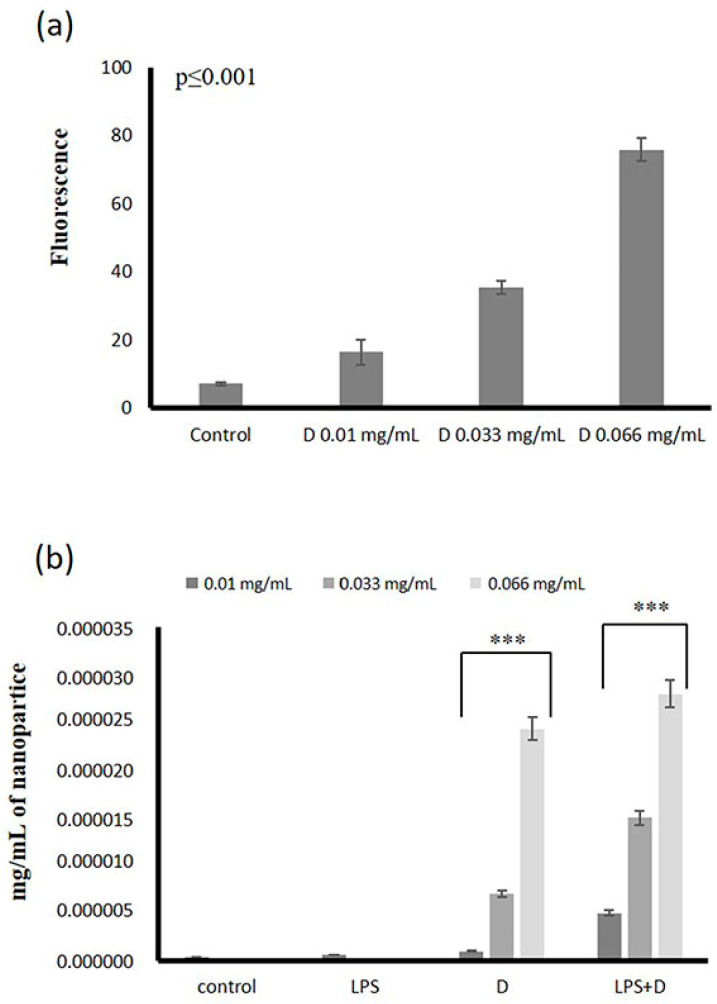
Uptake of DNPs by HMEC-1 cells. (**a**) Concentration-dependent fluorescence of DNPs; (**b**) uptake of DNPs (D) by normal and LPS-activated endothelial cells expressed as mg/mL of cell extracts. The cells were cultured in 96-well plates, in some cases activated with LPS, and treated with DNPs (D) for 48 h. The results represent the average of three separate experiments. Statistical significance: *** *p* ≤ 0.001 compared to respective controls.

The microvascular ECs are the first barrier NPs must traverse to reach the parenchyma [3]. NPs’ uptake is highly heterogenous and cell type-dependent [54]. Moreover, ECs of different organs were shown to exhibit different NP uptake efficiency, possibly attributed to variations in receptor expression, affinity, and activity [55]. Specificities in the uptake of NPs by ECs must be considered when developing targeted nanomedicine [55,56]. In this study, DNPs were diluted in PBS/cell culture medium, and the fluorescence of samples expressed as mg/mL was determined. A standard curve to verify the concentration-dependent intensity of fluorescence of diluted DNPs was generated, and fluorescence intensity was expressed as arbitrary units (AU) (Figure 6a) (*p* ≤ 0.001). The ECs were treated with various concentrations of DNPs for 48 h, after which the cells were harvested, and the fluorescence of cell extracts was assessed. This strategy determined a concentration-dependent uptake of DNPs by microvascular ECs (*p* ≤ 0.001) (Figure 6b). Subsequently, a model of activated endothelium was generated by treating the HMEC-1 cells with LPS for 2 h. The activated cells were then exposed to DNPs for 48 h; after that, they were harvested, cell extracts collected, and the fluorescence of respective samples determined. This approach demonstrated a dose-dependent increase in the uptake of DNPs (*p* ≤ 0.001, between samples) by activated microvascular ECs (Figure 6b). However, no differences were detected between the uptake of basal and activated endothelium (*p* = NP) (Figure 6b).

### 2.5. Uptake of p(VP-AA) NPs by Living HMEC-1 Cells

**Figure 7:**
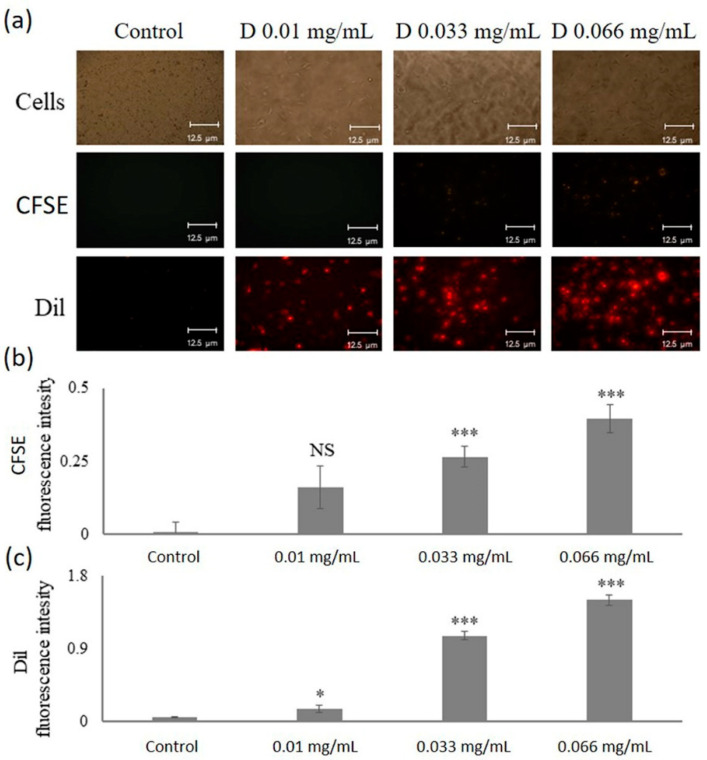
Detection of DNPs uptake by living HMEC-1 cells. HMEC-1 cells were seeded in 24-well plates at 150,000 cells/well and incubated in a complete MCDB medium for 24 h. The medium was replaced with a 2% FBS medium for 24 h. Then, HMEC-1 cells were incubated with different DNPs (D) concentrations (0.01 mg/mL, 0.033 mg/mL, 0.066 mg/mL) for 48 h in 2% FBS medium. Control cells were incubated only in a 2% FBS medium. (**a**) Concentration-dependent DNPs (D) uptake by living HMEC-1 cells was assessed using a fluorescence microscope (green; CFSE and red; Dil); magnification ×10. Representative images are presented. (**b**) Fluorescence intensity of CFSE dye. (**c**) Fluorescence intensity of Dil dye. Statistical significance: * *p* ≤ 0.05, *** *p* ≤ 0.001, NS = not significant compared to respective controls.

To visualize the uptake of DNPs by living HMEC-1 cells, DNPs, and fluorescence microscopy were utilized. The CFSE dye fluoresces with green, while the DiI dye fluoresces with red. As shown in Figure 7a, a concentration-dependent increase in the intracellular deposition of both CFSE and DiI dyes was evident, confirming the ability of HMEC-1 cells to uptake p(VP-AA) NPs in a concentration-dependent manner. Specifically for CFSE uptake, the statistical significance was *p* ≤ 0.001 for 0.033 and 0.066 mg/mL treatments against control, whereas for DiI, the statistical significance was *p* ≤ 0.05, *p* ≤ 0.001, *p* ≤ 0.001 for 0.010, 0.033, and 0.066 mg/mL treatments against control, respectively (Figure 7b and c).

### 2.6. Intracellular Localization of DNPs

**Figure 8:**
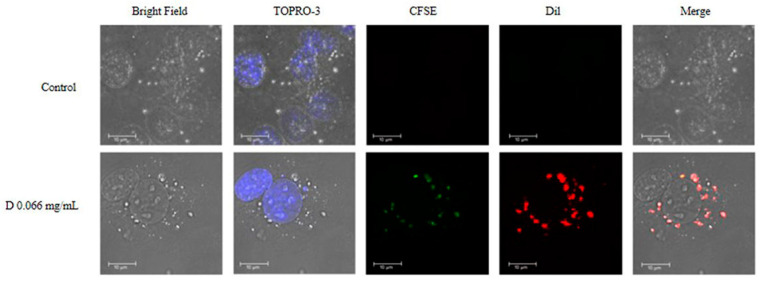
Intracellular localization of DNPs. HMEC-1 cells were seeded on round coverslips placed in 24-well plates at a concentration of 150,000 cells/well and incubated in a complete medium for 24 h. The media was replaced with a 2% FBS medium for 24 h. HMEC-1 cells were treated with 0.066 mg/mL of DNPs (CFSE; green stain, Dil; red stain) for 48 h at 37 °C and 5% CO_2_. The cells were fixed and stained with TO-PRO-3 iodide. The coverslips were then mounted onto slides using glycerol and visualized using confocal microscopy; magnification ×40, and zoom 6. Representative images are presented.

It is well established that cellular uptake is not only a key event for drug delivery but is also immediately correlated to the biological consequences of NPs utilization [3,24]. To observe the internalization and intracellular localization of NPs, HMEC-1 endothelial cells treated with the high dose (0.066 mg/mL) of DNPs were subsequently investigated with confocal microscopy. Figure 8 demonstrates diffuse staining of the CFSE dye (green stain) and the DiI dye (red color) in the cytoplasm of DNP-treated cells. The NPs are observed in endosomes within the cytoplasm. The cell nucleus was stained with TO-PRO- 3 (blue stain). Bright-field microscopy was also utilized to define cell boundaries. This approach showed that p(VP-AA) NPs are located in the cytoplasm but not in the nucleus of HMEC-1 cells. Other studies have also determined that upon uptake, PVP-based NPs are localized in the endosomes in the cytoplasm of endothelial cells [57]. Moreover, PVP NPs are suggested to be internalized through clathrin-mediated endocytosis [58].

### 2.7. Effect of p(PV-AA) NPs and DNPs on HMEC-1 Cells Adhesion Molecule Expression

The endothelium plays a prominent role in the inflammatory response [59]. Under inflammatory response conditions such as anaphylaxis or sepsis, intercellular contacts disintegrate in post-capillary venules resulting in intercellular gap formation-dependent edema and increased transmigration of immune cells to tissue parenchyma [60]. Furthermore, an increase in the expression of adhesion receptors, including E-selectin, ICAM-1, and V-CAM-1, facilitating leukocyte diapedesis and subsequent circulation of leukocytes is evident in various pathological conditions with an inflammatory component [61,62]. In an earlier study, we demonstrated that Amph-PVP NPs did not affect the activation of endothelial cells [11].

**Figure 9:**
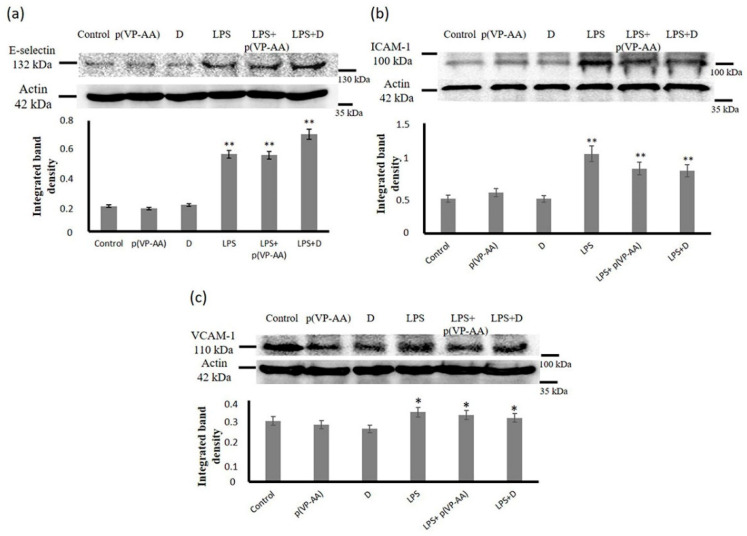
Effect of p(VP-AA) and DNPs on the expression of HMEC-1 cells adhesion molecules. (**a**) Effect of-p(VP-AA) NPs on E-selectin expression of basal state and LPS-activated HMEC-1 cells; (**b**) Effect of p(PV-AA) NPs on the expression of the ICAM-1 of basal state and activated HMEC-1 cells; (**c**) Effect of p(PV-AA) NPs on the expression of the VCAM of basal state and activated HMEC-1 cells. The results represent the average of three separate experiments. Statistical significance: * *p* ≤ 0.05, ** *p* ≤ 0.01, compared to respective controls.

To evaluate the effect of p(VP-AA) and DNPs on basal state and activated endothelium, the HMEC-1 cells were treated with the respective NPs or a combination of NPs and LPS at a concentration verified to induce these cells’ activation. Analysis of treated cell extracts showed that exposure to p(VP-AA) and DNPs did not affect the expression of ICAM-1, VCAM, and E-selectin adhesion molecules (*p* = NS) (Figure 9a–c). As expected, an increased expression of ICAM-1, VCAM, and E-selectin molecules upon LPS treatment was demonstrated (*p* ≤ 0.05) compared to control, whereas co-administration of non-labeled and labeled p(VP-AA) NPs did not enhance the LPS-dependent increase of the respective adhesion molecules (*p* = NS) (Figure 9a–c). These data, therefore, show that p(VP-AA) NPs do not affect the ECs’ expression of leukocyte adhesion molecules and do not contribute to processes of endothelial activation.

## 3. Materials and Methods

### 3.1. Materials

N-vinyl-2-pyrrolidone (VP), 2,2′-azobisisobutyronitrile (AIBN), 1,4-dioxane, and 6-hexane diamine was obtained from Acros Organics (Thermo Fisher Scientific, Geel, Belgium). Acrylic acid (AA), 5(6)-carboxyfluorescein diacetate N-succinimidyl ester (CFSE), 1,1′-dioctadecyl-3,3,3′,3′-tetramethylindocarbocyanine perchlorate (DiI), succinimide, N, N′-dicyclohexylcarbodiimide (DCC, 1.0 M solution in methylene chloride), octadecyl mercaptan (ODM), 1,6-diphenyl-1,3,5-hexatriene (DPHT), Fluorescein 5-isothiocyanate (CFSE), and 3-chloroperoxybenzoic acid, Dulbecco’s phosphate-buffered saline (DPBS), ethyl acetate, methanol, ethanol, dimethylsulfoxide (DMSO), phosphate buffer saline (PBS), (ethylene glycol-bis(β-aminoethyl ether)-N, N, N′, N′-tetraacetic acid) buffer (EGTA), indomethacin, and all other chemicals used in this study were purchased from Sigma-Aldrich (Sigma-Aldrich, St. Louis, MS, USA) unless otherwise specified and used without further purification. Analytical grade preparations were used for all solvents and buffer solution components. The Millipore Milli-Q plus System (Merck KGaA, Darmstadt, Germany) was used to prepare distilled-deionized water.

### 3.2. Synthesis of Amphiphilic Poly-N-Vinylpyrrolidone-Based Polymeric Materials

Specifically, an amphiphilic copolymer of N-vinyl-2-pyrrolidone and acrylic acid, namely, p(VP-AA), was synthesized and characterized as described in a previous study [22]. The polymer was purified by dialysis against water (Slide-A-LyzerDialysis Flask, 3K MWCO, Thermo Scientific, Waltham, MA, USA) and was lyophilized (BETA 2-8Ldplus, Martin Christ Gefriertrocknungsanlagen GmbH, Osterode am Harz, Germany) before subsequent modification. The CAC of the nanoparticles prepared from p(VP-AA) was determined by fluorescence spectroscopy (Hitachi High-Tech Corporation, model 650-10S, Tokyo, Japan) by using 1,6-diphenyl-1,3,5-hexatriene (DPHT) as the fluorescent probe.

### 3.3. Modification of p(VP-AA) by CFSE

The p(VP-AA) copolymer was modified by CFSE using carbodiimide chemistry. CFSE contains a maleimide group, and we used it to connect the dye to acrylic acid carboxyl groups of our polymer in NPs. For this, the copolymer was dissolved in chloroform, and the equivalent amount of succinimide and N, N′-di-cyclohexyl carbodiimide solution was added under stirring at room temperature (the mole ratio between carboxide groups: N, N′-di-cyclo hexyl carbodiimide: succinimide was 1:1.2:1.2). The solution was mixed for one hour. After this, the 6-hexane diamine was added, and the mixture was mixed additionally for 30 min. Finally, the mixture containing p(VP-AA)-OD copolymer with active amine groups was purified by dialysis against water and lyophilized.

The p(VP-AA) copolymer contained reactive amine groups dissolved in chloroform and the solution of CFSE in chloroform (the mole ratio between initial carboxide groups: CFSE was 1:1.2). The solution was mixed at room temperature for one hour and then purified by dialysis against water and lyophilized. Thus, the p(VP-AA) linked with the CFSE fluorophore marker was obtained. The concentration of linked CFSE was determined by UV spectroscopy (Unico SQ-2802PCS UV–Vis spectrophotometer, Thermofisher, Dreieich, Germany). The morphology of the Amph-PVP nanoparticles was determined by transmission electronic microscopy (TEM), using a JEM-2100 microscope (JEOL, Peabody, MA, USA).

### 3.4. Preparation and Characterization of p(VP-AA) NPs Carrying Fluorophores

DNPs were prepared by using an emulsification method. More specifically, appropriate amounts of the p(VP-AA) polymer were dispersed in a specific volume of H_2_O, and particular quantities of DiI were dissolved in the organic solvent, in this case, chloroform. This was followed by an oil-in-water (O/W) emulsification, solvent evaporation, and freeze-drying.

The CAC was determined by fluorescence spectroscopy (Hitachi High-Tech Corporation,, model 650-10S, Tokyo, Japan) using 1,6-diphenyl-1,3,5-hexatriene (DPHT) as the fluorescent probe. The particle size distribution and ζ-potential were determined by the dynamic light scattering (DLS) method (Malvern Panalytical Ltd., Zetasizer Nano ZS, Malvern, UK). Polymer synthesis and NP preparation are fully described in our previous works. Here we used the same methods [11,22].

### 3.5. Cell Culture

HMEC-1 human dermal microvascular endothelial cell line was utilized in the current study. Cells were grown in MCDB 131 (Biochrom AG; F0455) supplemented with 10% fetal bovine serum (FBS; Invitrogen 10500-064; heat inactivated), glutamine (10 mM; Biosera XCT1715), hydrocortisone (1 μg/mL), gentamycin (Invitrogen; 15710-049), and penicillin/streptomycin (100 units/mL; Biosera LMA4118). Before adding treatments, cells were cultured in a 2% medium for 24 h at 37 °C and 5% CO_2_.

### 3.6. Fluorescence Microscopy

HMEC-1 cells were seeded in 24-well plates at 150,000 cells/well concentration and incubated in a complete medium for 24 h. The medium was replaced with a 2% FBS medium for 24 h. Treatments were added and the cells were incubated for 48 h at 37 °C and 5% CO_2_. The medium was removed; the cells were washed twice with 2% FBS medium and replaced with a fresh one. Pictures were obtained using a fluorescent microscope (Leica, DM IRE2). The fluorescence intensity of CFSE and DiI was measured in different treatment concentrations by analyzing the pictures with the ImageJ Software. The signal intensity was measured and the background intensity was removed from the final value.

### 3.7. Measurement of Nanoparticle Uptake Utilizing Fluorescence

Growing cells were harvested and seeded in black 96-well plates (Corning; 3603) at a density of 3500 cells per well in 200 µL of MCDB 131 (10% FBS) for 24 h. Then, the medium was replaced with 200 µL of MCDB 131 (2% FBS) for 24 h. Treatments were added for the next 48 h at 37 °C and 5% CO_2_ in a 2% FBS medium. According to the manufacturer’s instructions, a standard curve of nanoparticle dilutions was calculated using the CyQUANT fluorometric assay (Thermo Scientific; C7026) and converted fluorescence units to mg/mL. Fluorescence was measured in a Fluorometer (Biotek, Winooski, VT, USA) using the proposed excitation (485 nm) and emission filters (528 nm).

### 3.8. Confocal Microscopy

HMEC-1 cells were seeded on round coverslips placed in 24-well plates at a concentration of 150,000 cells/well and incubated in a complete medium for 24 h. The medium was replaced with a 2% FBS medium for 24 h. Treatments were added, and the cells were incubated for 48 h at 37 °C and 5% CO_2_. The cells were fixed with 5% formaldehyde and 2% sucrose in PBS for 10 min at RT. After three washes with PBS, the permeabilizing agent Triton X100 was added for 10 min. TO-PRO-3 iodide (Molecular Probes; T3605) diluted 1:500 in de-ionized H2O was applied for 40 min to stain nuclei. The coverslips were then mounted onto slides using glycerol and visualized using confocal microscopy (Leica, TCS SP2 SE).

### 3.9. MTT Assay

Growing cells were harvested and seeded in 96-well plates (Corning; 3603) at a density of 15,000 cells per well in 200 µL of MCDB 131 (10% FBS) for 24 h. Then, the medium was replaced with 200 µL of MCDB 131 (2% FBS) for 24 h. Finally, treatments were added for the next 48 h at 37 °C and 5% CO_2_ in a 2% FBS medium. Labeling of the cells and the measurements were performed according to the manufacturer’s instructions (ThermoFisher, Waltham, MA, USA, VybrantTmΜΤΤ Assay).

### 3.10. Cell Growth Assay

The growth of human microvascular endothelial HMEC-1 cells was assessed, as previously reported [11,14]. In short, HMEC-1 growing cells from non-confluent cultures were harvested and seeded in black 96-well plates (Corning; 3603) at a density of 3000 cells per well in 200 μL of MCDB 131 (10% FBS). The cell density number was chosen from optimization experiments. The cells were allowed to rest overnight. Treatments were added for the next 48 h at 37 °C and 5% CO_2_ in 0% FBS. The cells were then lysed, and their number was calculated using the CyQUANT fluorometric assay (Thermo Scientific; C7026) according to the manufacturer’s instructions. Fluorescence was measured in a Fluorometer (Biotek, Winooski, VT, USA) using the proposed excitation (485 nm) and emission (528 nm) filters. A separate standard curve was used to convert fluorescence units to cell numbers. All experiments were performed in triplicate.

### 3.11. Western Blot

Harvested cells were lysed with RIPA solution (50 mM Tris-HCl, 1% NP-40, 0.25% Na-Deoxycholate, 150 mM NaCl, 1 mM EDTA with protease and phosphatase inhibitors). Equal amounts of protein were subjected to SDS-PAGE using 10% polyacrylamide gels under reducing conditions. Separated protein bands were transferred to nitrocellulose membranes in 10 mM (pH 11), containing 10% methanol. Membranes were blocked overnight at 4 °C with PBS containing 0.1% Tween-20 (PBS-Tween) and 5% (*w*/*v*) low-fat milk powder. The membranes were incubated for 1 h at room temperature (RT) with the primary antibody in PBS containing 0.1% Tween-20 (PBS-Tween) and 1% (*w*/*v*) low-fat milk powder. The immune complexes were detected after incubation with the appropriate peroxidase-conjugated secondary antibody diluted (1:5000) in PBS-Tween, 2% low-fat milk, using the LumiSensor Chemiluminescent HRP substrate kit (Genscript; L00221V500), according to the manufacturer’s instructions. Protein expression of actin was used to correct the amount of each sample analyzed.

### 3.12. Statistical Analysis

The Student’s *t*-test or ANOVA analysis evaluated the statistical significance.

## 4. Conclusions

Ever-accumulating evidence introduces the beneficial utilization of NPs in biomedicine to enhance the specialized targeting of the desired tissues while reducing patient side effects [63,64].

To examine the interaction of amphiphilic poly-N-vinylpyrrolidone-based polymeric materials with endothelial cells, we synthesized a copolymer of PVP with acrylic acid and p(VP-AA) NPs. Furthermore, we labeled the NPs co-utilizing CFSE and DiI (DNPs). DNPs incorporated DiI molecules into their cores and linked CFSE molecules onto their surface. This approach enabled the determination of p(PV-AA) NPs uptake and intracellular localization in living cells. The uptake was found to be efficient and concentration dependent. Furthermore, p(VP-AA) and DNPs inhibited these cells’ viability at higher concentrations in a similar, concentration-dependent manner. Generating a model of activated endothelium demonstrated that p(VP-AA) and DNPs decreased ECs’ viability, identical to the basal state conditions. Likewise, the growth of ECs was attenuated upon exposure to high concentrations of p(VP-AA) and DNPs. The concentrations at which p(VP-AA) NPs exert adverse effects are much higher than the normal blood plasma therapeutic range of a vast majority of drugs [65]. Since NPs are utilized as drug carriers, it would be difficult to predict their needed concentration without performing pharmacokinetic studies for a specific drug. P(PV-AA) NPs are shown in this study to be well tolerated by endothelial cells at concentrations higher than plasma therapeutical levels of most drugs. Therefore, we can extrapolate that, at least in the in vitro model, they do not adversely affect cell viability at pharmacologically relevant concentrations. Moreover, p(VP-AA) and DNPs did not affect the expression of adhesion receptors, including E-selectin, ICAM-1, and V-CAM, which facilitate leukocyte diapedesis and their subsequent infiltration to surrounding tissue.

The synthesis of fluorescent NPs demonstrated that CFSE- and DiI-labeling did not change the biocompatibility profile of p(VP-AA). To sum up, p(VP-AA) NPs exert adverse effects on Ecs’ viability and growth concentrations much higher than the normal therapeutic range of most drugs and are non-inflammatory at all concentrations tested. CFSE and DiI labeling is efficient, safe, and allows better evaluation of p(VP-AA) interactions with cells and tissues. Further study of p(VP-AA) NPs interactions with biological systems are in order.

## Figures and Tables

**Table 1:** Characteristics of hollow p(VP-AA) and labeled DNPs.

NPs	Particle Size (nm)	PDI	Zeta Potential (mV)
Hollow p(VP-AA)	155 ± 8	0.174 ± 0.025	−17.9 ± 0.91
DiI loaded p(VP-AA) linked with CFSE nanoparticles (DNPs)	172 ± 10	0.156 ± 0.031	−15.2 ± 1.14

(mean ± SD, n = 3) in PBS at 37 °C.

## Data Availability

Not applicable.

## Supplementary Materials

The supporting information can be downloaded at: https://www.mdpi.com/article/10.3390/ijms232012446/s1.
