# Supplementary material for: In Vitro Assessment of Poly-N-Vinylpyrrolidone/Acrylic Acid Nanoparticles Biocompatibility in a Microvascular Endothelium Model
Source: Int J Mol Sci. 2022 Oct 18;23(20):12446. doi: 10.3390/ijms232012446 (PMC9604021; doi:10.3390/ijms232012446)
Supplement: Supplementary file 1 [file ijms-23-12446-s001.zip › ijms-1922551-supplementary.pdf]

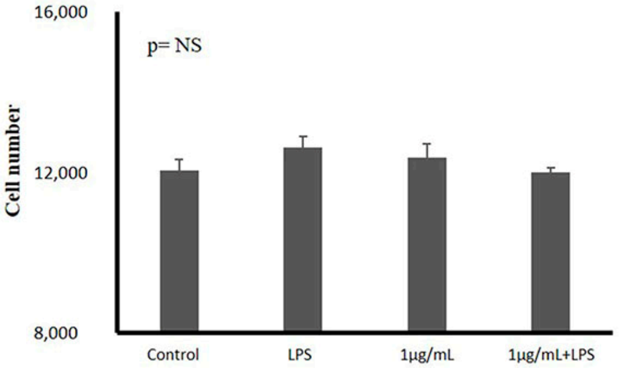

Figure S1. Effect of p(VP-AA) on the viability of basal and activated HMEC-1 cells. Effect of low concentration p(VP-AA) NPs (0.001 mg/mL) on HMEC-1 cells' viability. Basal state and exposed to LPS cells were cultured in 96-well plates and treated with p(VP-AA) NPs for 48 hours. The results represent the average of three separate experiments. Statistical significance: NS = not significant.

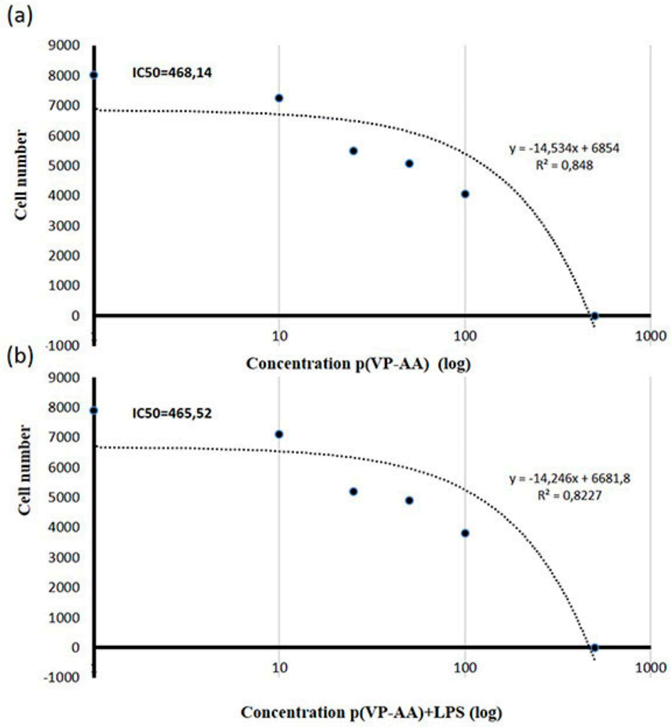

Figure S2. Calculation of IC50 for p(VP-AA) concentration in the viability of HMEC-1 cells. Basal state (**a**) and exposed to LPS (**b**) cells were cultured in 96-well plates and treated with p(VP-AA) concentrations 1, 10, 25, 50, 100, 500 mg/mL, for 48 hours. IC50 was calculating using the graphs' equation and y = 50.
